# Hyperthyroidism-Induced Dural Venous Sinus Thrombosis: A Case Report

**DOI:** 10.7759/cureus.102166

**Published:** 2026-01-23

**Authors:** Anas E Ahmed, Abdulrahman K Alghamdi, Yasmin A Alghamdi, Jannat K Aljohani, Ghada G Almaziad

**Affiliations:** 1 Community Medicine, Jazan University, Jazan, SAU; 2 Medicine, Universiti Sains Malaysia, Kelantan, MYS; 3 Medicine, King Abdulaziz University, Jeddah, SAU; 4 Medicine, King Saud Bin Abdulaziz University for Health Sciences, Riyadh, SAU

**Keywords:** anticoagulation, cerebral venous sinus thrombosis, dural venous sinus thrombosis, graves’ disease, hyperthyroidism, neuroimaging, prothrombotic state, thyrotoxicosis, transverse sinus thrombosis

## Abstract

Cerebral dural venous sinus thrombosis is an uncommon and potentially life-threatening cerebrovascular disorder with a broad and often nonspecific clinical spectrum, frequently leading to diagnostic delay. We describe a young woman with no prior comorbidities who presented with a subacute history of progressively worsening headache associated with visual disturbances and pulsatile tinnitus. Neuroimaging with computed tomography and magnetic resonance venography demonstrated acute thrombosis of the right transverse dural venous sinus without parenchymal infarction or hemorrhage. Comprehensive laboratory evaluation revealed overt thyrotoxicosis with positive thyroid autoantibodies consistent with Graves’ disease, while an extensive thrombophilia work-up failed to identify alternative prothrombotic risk factors. The patient was treated with therapeutic anticoagulation alongside prompt initiation of antithyroid therapy and beta-adrenergic blockade, leading to progressive symptom resolution, biochemical improvement toward euthyroidism, and radiological evidence of sinus recanalization on follow-up. This case emphasizes hyperthyroidism as an underrecognized yet reversible cause of cerebral venous sinus thrombosis and highlights the importance of systematic etiological evaluation, including thyroid function assessment, in patients with cerebral venous thrombosis to enable early targeted therapy and favorable neurological outcomes.

## Introduction

Cerebral dural venous sinus thrombosis (DVST) is an uncommon but potentially life-threatening cause of cerebrovascular disease, accounting for less than 1% of all strokes [1,2]. It predominantly affects younger individuals and has a wide spectrum of clinical presentations, ranging from isolated headache to focal neurological deficits, seizures, and altered consciousness. The condition results from thrombosis of the intracranial venous sinuses, leading to impaired venous drainage, raised intracranial pressure, venous infarction, or hemorrhage [2-4]. Numerous risk factors have been identified, including inherited and acquired thrombophilias, pregnancy and the puerperium, malignancy, infection, dehydration, and use of oral contraceptives [3,5]. Despite advances in neuroimaging facilitating early diagnosis, DVST remains a diagnostic challenge due to its nonspecific and variable presentation.

Hyperthyroidism is an established but underrecognized prothrombotic state. Excess thyroid hormones are known to induce a hypercoagulable and hypofibrinolytic milieu through increased levels of clotting factors, reduced fibrinolysis, endothelial dysfunction, and enhanced platelet activation [2-4]. Graves’ disease, the most common cause of hyperthyroidism, has been increasingly associated with venous thromboembolic events, including deep vein thrombosis (DVT), pulmonary embolism (PE), and, more rarely, cerebral venous sinus thrombosis [3,4]. Awareness of hyperthyroidism as a reversible risk factor for DVST is crucial, as prompt recognition and simultaneous management of both conditions can significantly improve outcomes. This case highlights the rare association between thyrotoxicosis and dural venous sinus thrombosis involving the transverse sinus, underscoring the importance of considering endocrine disorders in the etiological evaluation of cerebral venous thrombosis.

## Case presentation

A 32-year-old woman with no significant past medical history presented to the emergency department with a 10-day history of progressively worsening headache. The headache was described as constant, pressure-like, predominantly occipital with right-sided predominance, and associated with intermittent nausea and photophobia. Over the preceding three days, she also noted transient episodes of blurred vision and pulsatile tinnitus in the right ear. There was no history of trauma, fever, neck stiffness, seizures, focal limb weakness, or loss of consciousness. On further questioning, she reported a three-month history of unintentional weight loss of approximately 8 kg despite increased appetite, heat intolerance, excessive sweating, palpitations, anxiety, and fine tremors of the hands. She denied prior thromboembolic events, recent surgery, prolonged immobilization, oral contraceptive use, pregnancy, or miscarriage. There was no personal or family history of thrombophilia or autoimmune disease.

On physical examination, the patient was alert and oriented, appearing anxious but in no acute distress. Vital signs revealed a blood pressure of 128/76 mmHg, heart rate of 118 beats per minute with regular rhythm, respiratory rate of 18 breaths per minute, temperature of 36.8°C, and oxygen saturation of 99% on room air. General examination showed a fine tremor of outstretched hands, warm, moist skin, and mild proximal muscle weakness. Thyroid examination revealed a diffusely enlarged, non-tender thyroid gland without palpable nodules or bruit. Ophthalmologic examination demonstrated mild bilateral lid retraction without proptosis; fundoscopic examination showed no papilledema. Cardiovascular examination revealed tachycardia without murmurs, while respiratory and abdominal examinations were unremarkable. Neurological examination showed intact cranial nerves, normal motor strength and sensation in all extremities, and no cerebellar signs or meningeal irritation.

Initial laboratory investigations demonstrated suppressed thyroid-stimulating hormone (TSH) (<0.01 mIU/L), elevated free thyroxine (FT4) and free triiodothyronine (FT3) levels, consistent with overt hyperthyroidism. Thyroid peroxidase antibodies and thyrotropin receptor antibodies were positive, supporting a diagnosis of Graves’ disease. Complete blood count revealed mild leukocytosis, while hemoglobin and platelet counts were within normal limits. Serum electrolytes, renal function tests, and liver function tests were normal. Coagulation profile showed a mildly elevated D-dimer level, with normal prothrombin time and activated partial thromboplastin time. A comprehensive thrombophilia work-up, including protein C, protein S, antithrombin III levels, antiphospholipid antibodies, factor V Leiden mutation, and prothrombin gene mutation, was unremarkable (Table 1).

**Table 1:** Summary of laboratory investigations at presentation and during the diagnostic workup Reference ranges correspond to standard adult values.

Laboratory parameter	Value	Unit	Reference range
Hemoglobin	12.8	g/dL	12.0-15.5
Hematocrit	38.5	%	36-46
White blood cell count	11.6	×10⁹/L	4.0-10.0
Neutrophils	72	%	40-75
Lymphocytes	22	%	20-45
Platelet count	310	×10⁹/L	150-400
C-reactive protein	4.2	mg/L	<5.0
Erythrocyte sedimentation rate	18	mm/hr	<20
Serum creatinine	0.72	mg/dL	0.6-1.1
Blood urea nitrogen	14	mg/dL	7-20
Alanine aminotransferase	28	U/L	7-35
Aspartate aminotransferase	26	U/L	8-40
Alkaline phosphatase	112	U/L	44-147
Total bilirubin	0.8	mg/dL	0.2-1.2
Albumin	4.2	g/dL	3.5-5.0
Sodium	138	mmol/L	135-145
Potassium	4.1	mmol/L	3.5-5.1
Chloride	102	mmol/L	98-107
Calcium (corrected)	9.6	mg/dL	8.5-10.5
Prothrombin time	12.1	seconds	10-13
International normalized ratio	1.0	—	0.8-1.2
Activated partial thromboplastin time	29	seconds	25-35
D-dimer	1.12	mg/L FEU	<0.5
Protein C activity	98	%	70-140
Protein S activity	92	%	60-130
Antithrombin III	104	%	80-120
Lupus anticoagulant	Negative	—	Negative
Anticardiolipin antibodies (IgG/IgM)	Negative	—	Negative
Factor V Leiden mutation	Not detected	—	—
Prothrombin G20210A mutation	Not detected	—	—
Thyroid-stimulating hormone	<0.01	mIU/L	0.4-4.5
Free thyroxine	3.6	ng/dL	0.8-1.8
Free triiodothyronine	9.2	pg/mL	2.3-4.2
Thyrotropin receptor antibody	6.8	IU/L	<1.75
Thyroid peroxidase antibody	420	IU/mL	<35

**Figure 1:**
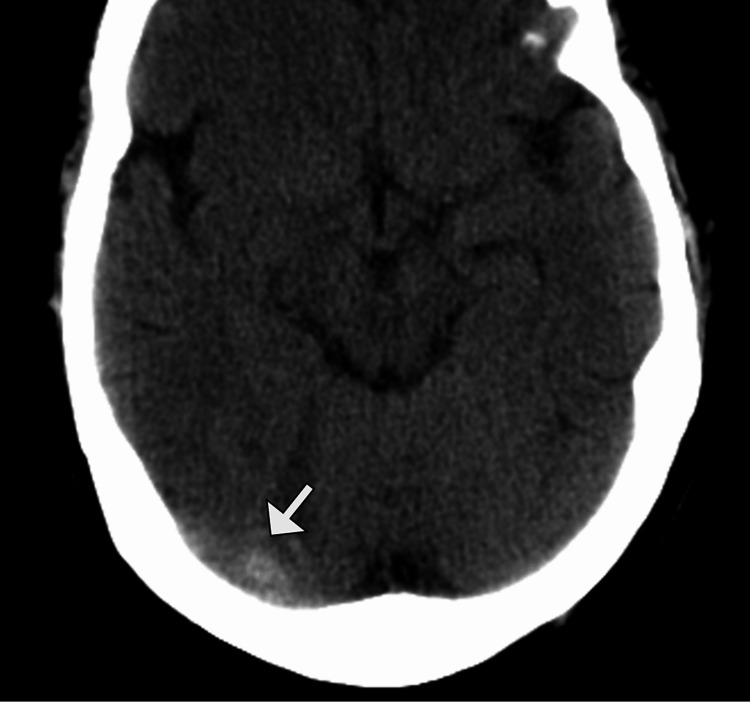
Noncontrast CT demonstrating right transverse sinus thrombosis Axial noncontrast computed tomography (CT) image of the brain shows a focal hyperdensity within the right transverse sinus (arrow), consistent with the dense sinus sign and suspicious for acute transverse sinus thrombosis.

Given the persistent headache and visual symptoms, a non-contrast computed tomography (CT) scan of the brain was performed and showed hyperdensity along the right transverse sinus, raising suspicion for DVST. There was no evidence of intracerebral hemorrhage or mass effect (Figure 1).

**Figure 2:**
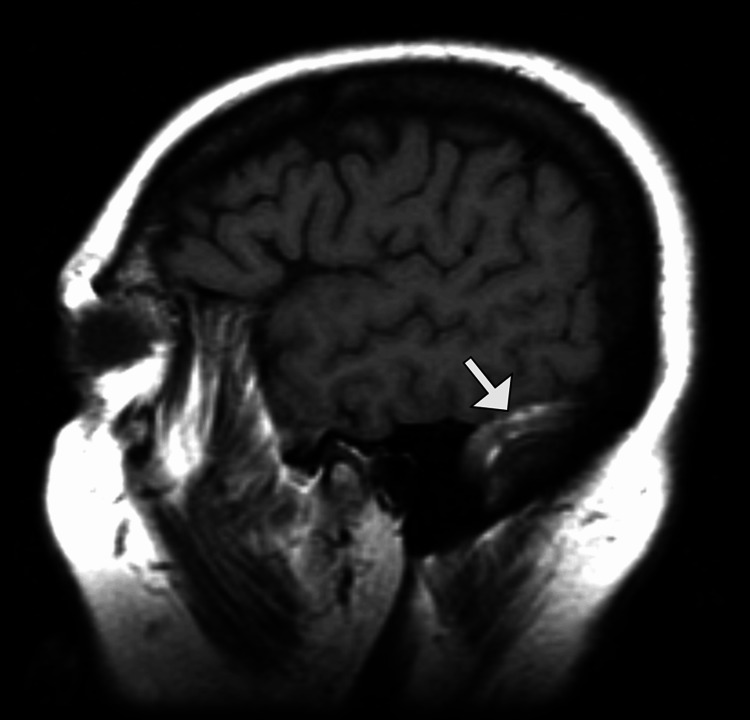
T1-weighted MRI showing thrombus within the transverse sinus Sagittal T1-weighted magnetic resonance imaging (MRI) demonstrates abnormally high signal intensity within the right transverse sinus (arrow), compatible with a subacute thrombus.

Subsequent contrast-enhanced magnetic resonance imaging (MRI) of the brain confirmed the absence of a normal flow void and a filling defect in the right transverse sinus, consistent with acute DVST (Figure 2).

**Figure 3:**
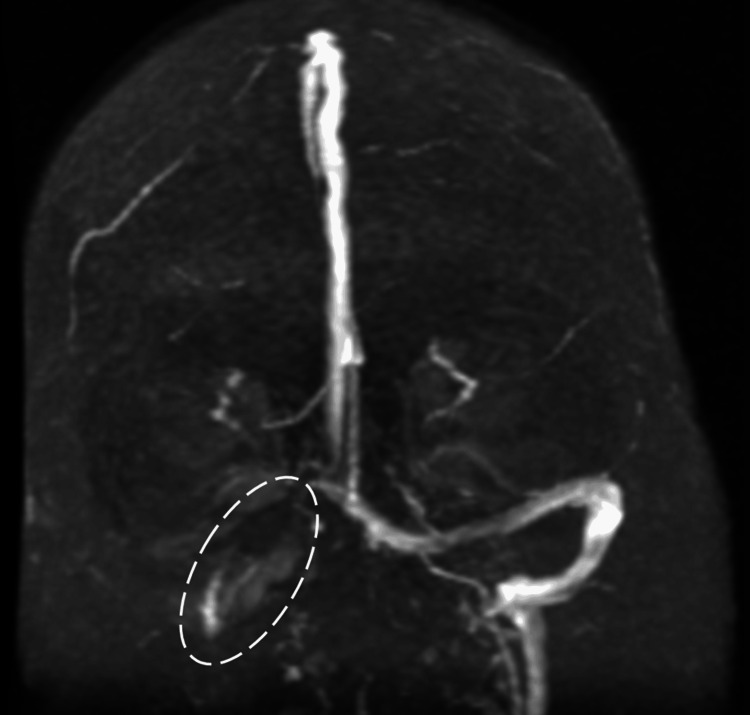
Time-of-flight MR venography demonstrating transverse sinus occlusion Three-dimensional (3D) time-of-flight (TOF) magnetic resonance (MR) venography shows a filling defect within the right transverse sinus (encircled), confirming the presence of transverse sinus thrombosis.

The remaining dural sinuses and deep venous system were patent, and there were no associated venous infarcts or hemorrhagic lesions (Figure 3).

The presence of biochemical thyrotoxicosis, supportive imaging, and exclusion of other thrombophilic conditions led to the diagnosis of hyperthyroidism-induced DVST involving the right transverse sinus.

The patient was admitted to the neurology service and initiated on therapeutic anticoagulation with low-molecular-weight heparin (LMWH), later transitioned to oral anticoagulation. Concurrently, management of hyperthyroidism was started with methimazole and a non-selective beta-blocker to control adrenergic symptoms. Adequate hydration and analgesia were provided, and close neurological monitoring was maintained. No signs of neurological deterioration or bleeding complications were observed during hospitalization.

Over the subsequent days, the patient reported gradual improvement in headache intensity and resolution of visual disturbances. Heart rate normalized with beta-blockade, and thyroid hormone levels began to trend downward. She remained neurologically intact throughout her hospital stay. After seven days of inpatient management, she was discharged in stable condition on oral anticoagulation, antithyroid medication, and beta-blocker therapy, with instructions for close outpatient follow-up.

At follow-up six weeks later, the patient was asymptomatic with no recurrence of headache or neurological complaints. Thyroid function tests showed significant improvement toward euthyroidism. Anticoagulation was planned to be continued for a total duration of six months, with ongoing endocrinology and neurology follow-up to monitor thyroid status and venous sinus patency.

## Discussion

Cerebral DVST is a rare and heterogeneous cerebrovascular disorder with multifactorial etiology, often posing diagnostic and therapeutic challenges. The present case highlights an uncommon but clinically significant association between hyperthyroidism and DVST, specifically involving the transverse sinus, and contributes to the growing body of evidence supporting thyrotoxicosis as an independent prothrombotic state. Although headache remains the most frequent presenting symptom of DVST, its nonspecific nature frequently leads to delayed diagnosis [4-6]. In this patient, the coexistence of progressive headache with subtle visual symptoms and systemic features of hyperthyroidism underscores the importance of comprehensive clinical assessment and early neuroimaging in suspected cases.

The pathophysiological link between hyperthyroidism and venous thrombosis is complex and multifaceted. Excess thyroid hormones exert profound effects on the hemostatic system, promoting a hypercoagulable and hypofibrinolytic state [5,6]. Elevated levels of coagulation factors such as factor VIII, fibrinogen, von Willebrand factor, and factor IX, along with reduced fibrinolytic activity due to increased plasminogen activator inhibitor-1, have been well documented in thyrotoxic patients [4-8]. Additionally, thyroid hormones enhance platelet activation and aggregation and may induce endothelial dysfunction, further predisposing to thrombus formation. These mechanisms collectively explain the increased risk of venous thromboembolism observed in hyperthyroid states, even in the absence of inherited or acquired thrombophilia, as demonstrated by the negative thrombophilia work-up in this case [3,8].

Graves’ disease has been particularly implicated in thromboembolic complications. Autoimmune-mediated endothelial injury and systemic inflammation may further amplify the prothrombotic risk in these patients [2-4]. While DVT and PE are more frequently reported, cerebral venous sinus thrombosis remains a rare manifestation, with the transverse and superior sagittal sinuses most commonly involved. The relative rarity of DVST in hyperthyroidism may contribute to underrecognition, emphasizing the need for heightened clinical awareness [5,6]. This case reinforces the notion that thyroid function testing should be considered in the etiological evaluation of DVST, especially in younger patients without conventional risk factors.

Neuroimaging plays a pivotal role in the diagnosis of DVST. Non-contrast CT, though readily available, may be normal or show subtle indirect signs, such as sinus hyperdensity, as seen in this patient [3,5]. MRI combined with magnetic resonance venography (MRV) remains the gold standard, allowing direct visualization of thrombus, assessment of venous flow, and identification of associated parenchymal complications. Early and accurate diagnosis is essential, as prompt treatment significantly improves outcomes and reduces the risk of long-term neurological sequelae [5-7].

Anticoagulation remains the cornerstone of DVST management, even in the presence of hemorrhagic venous infarction, given its proven efficacy in preventing thrombus propagation and facilitating recanalization. In hyperthyroidism-associated DVST, simultaneous treatment of the underlying endocrine disorder is equally critical [2-8]. Restoration of euthyroidism has been shown to reverse the hypercoagulable state and may reduce the risk of recurrent thrombosis. The favorable clinical and radiological outcome observed in this patient highlights the effectiveness of combined anticoagulation and antithyroid therapy. However, the optimal duration of anticoagulation in this subset of patients remains uncertain, with most experts advocating treatment analogous to provoked venous thromboembolism, typically for three to six months, tailored to individual risk factors and follow-up imaging findings [4-7].

## Conclusions

Cerebral DVST is an uncommon but potentially life-threatening cause of cerebrovascular disease, predominantly affecting younger individuals. Hyperthyroidism is an established but underrecognized prothrombotic state. Graves’ disease has been increasingly associated with venous thromboembolic events, including DVT, PE, and, more rarely, cerebral venous sinus thrombosis. Awareness of hyperthyroidism as a reversible risk factor for DVST is crucial, as prompt recognition and simultaneous management of both conditions can significantly improve outcomes. This case highlights the rare association between thyrotoxicosis and dural venous sinus thrombosis involving the transverse sinus, underscoring the importance of considering endocrine disorders in the etiological evaluation of cerebral venous thrombosis.
